# Vaccination Coverage for Medically Indicated Vaccines in a Convenience Sample of Severely Immunocompromised Patients with COVID-19: An Observational Cohort Study

**DOI:** 10.3390/vaccines12121383

**Published:** 2024-12-09

**Authors:** Elsemieke te Linde, Marjolein P. M. Hensgens, Albert M. Vollaard, Annelies Verbon, Anke H. W. Bruns

**Affiliations:** 1 Department of Infectious Diseases, University Medical Centre Utrecht, 3584 CX Utrecht, The Netherlands; m.p.m.hensgens@umcutrecht.nl (M.P.M.H.); a.verbon@umcutrecht.nl (A.V.); a.h.w.bruns@umcutrecht.nl (A.H.W.B.); 2 Centre for Infectious Disease Control (CIb), National Institute for Public Health and the Environment (RIVM), 3721 MA Bilthoven, The Netherlands; albert.vollaard@rivm.nl

**Keywords:** vaccination coverage, immunization schedule, medically indicated vaccines, immunocompromised patients

## Abstract

Background: In recent decades, the number of immunocompromised patients (ICPs) has increased significantly. ICPs have an impaired immune system, making them susceptible to complicated infections. To protect them from infections, ICPs are eligible to receive several medically indicated vaccines. To obtain insight into the uptake of these medically indicated vaccines, we determined the coverage of these vaccines in ICPs. Methods: This observational cohort study was conducted at the University Medical Centre Utrecht, the Netherlands, from September 2021 to April 2022. All adult ICPs admitted for COVID-19 were asked to complete a questionnaire on their vaccination history (pneumococcal, herpes zoster, human papillomavirus vaccination, influenza, and COVID-19 vaccines) and history of vaccine-preventable infections. In addition, patients’ vaccination history was reviewed in medical files. Results: A total of 115 patients completed the questionnaire and were included. Although all patients had an indication for pneumococcal vaccination, only 22 received it (19%). Coverage for herpes zoster was low (1%, 1/106 eligible patients). Coverage for human papillomavirus vaccination (HPV) was also low (40%, two out of five eligible patients). In contrast, 92% of patients received vaccination against SARS-CoV-2, and 77% of patients received seasonal influenza vaccination. Conclusions: Although coverage for influenza and COVID-19 vaccination was high in ICPs, coverage for other medically indicated vaccines was low. Identifying which factors contributed to high COVID-19 and influenza vaccine uptake can help to improve vaccination rates for the other recommended vaccines. Clear guidelines for clinicians and the removal of organizational obstacles are needed to improve vaccination coverage.

## 1. Introduction

Immunocompromised patients (ICPs) are more susceptible to infection and face a higher risk of a complicated course than immunocompetent persons. To mitigate this increased risk, additional vaccinations are recommended for ICPs. Optimization of vaccination coverage for vaccine-preventable diseases is therefore crucial. Recommended vaccines for ICPs include those for pneumococcal infections, herpes zoster, human papillomavirus (HPV), influenza, and measles. 

A large group of ICPs is represented by patients treated for autoimmune diseases, and the burden of these conditions continues to increase over time [1]. Furthermore, the incident cases of hematological malignancies have been increasing globally for decades [2]. Over 6% of the population is currently estimated to be immunosuppressed, making infection prevention for these patients critical [3]. 

Infection prevention through vaccination is increasingly recommended for ICPs. These recommendations may be guided by the growing understanding of vaccine immunogenicity in ICPs. This immunogenicity largely depends on two factors. First, the degree of immunosuppression. In more severe ICPs, such as patients with solid organ transplants, vaccine immunogenicity seems less [4,5]. Secondly, it depends on the vaccine type. 

Modern vaccines, such as mRNA vaccines, are more effective. Large-scale studies on COVID-19 vaccines have shown better immune responses in ICPs than initially expected [6,7]. With additional booster doses, their humoral vaccine responses approached those seen in healthy controls [8,9]. Additionally, they seem to be safe for administration in immunocompromised individuals and have not been shown to cause flares in patients with immune-mediated inflammatory diseases or rejection in patients with solid organ transplants [10,11,12,13].

Vaccination with the recombinant zoster vaccine (RZV) and the quadrivalent HPV vaccine has also demonstrated relatively good immune responses in various subgroups of ICPs. The vaccine response rates were 66–96% for RZV and 53–82% for the HPV vaccine across different immunocompromised groups [14,15,16]. As a result, recommendations for medically indicated vaccines are increasingly incorporated into disease-specific and national vaccination guidelines [17,18,19,20,21,22]. 

Despite these updated recommendations, studies in subgroups of ICPs indicate low vaccination coverage [23,24,25]. This low coverage has been attributed to various patient-related factors, such as distrust in vaccine efficacy and concerns about vaccination risks. Also, healthcare professional-related factors contribute to this low coverage, including unclear guidelines, lack of knowledge, unawareness of vaccination indications, provider hesitancy, time constraints, and reimbursement issues [26,27]. 

Previous studies on vaccination coverage in ICPs have mainly focused on those with inflammatory bowel disease (IBD) or rheumatoid arthritis, who are usually only moderately immunocompromised. Less research has been conducted on vaccination rates in patients who are severely immunocompromised [23,24]. Therefore, we aimed to determine the vaccination coverage of medically indicated vaccines in patients with predominantly severe immunocompromising conditions across various medical specialties. The results will give insight into current vaccination coverages in ICPs. This information can help improve strategies to increase vaccination coverage and reduce infections and related complications. 

## 2. Materials and Methods

### 2.1. Patient Selection

We performed an observational cohort study between September 2021 and April 2022 in the University Medical Centre Utrecht, the Netherlands. We used a convenience sample of adult ICPs who were admitted because of COVID-19 and were able to provide informed consent. A severe immunocompromised state was defined when one of the following conditions was present: any previous solid organ transplantation, recent (<2 years before inclusion) hematological stem cell transplantation, active hematological malignancy, primary immunodeficiency disorder, HIV infection with a CD4 count of less than 200/mm^3^, or a solid malignancy for which chemotherapy within the last 4 weeks and/or last immune therapy being within 3 months before inclusion [28]. Patients with chronic immune-mediated inflammatory diseases who receive immunosuppressive therapy were considered severely immunocompromised when using targeted therapy (i.e., JAK-inhibitors), biologic immune modulators (i.e., B-cell targeted therapies, TNF inhibitors, IL inhibitors) or when using non-biological immune modulators in combination therapy. Singular use of non-biological immune modulators was considered moderately immunocompromised [28,29]. Finally, patients taking prednisone were only considered immunocompromised if they used more than 10 mg/day or a cumulative dose of >700 mg [30]. For azathioprine, the criterion was more than 3.0 mg/kg/day, and for 6-mercaptopurine, more than 1.5 mg/kg/day [18]. Ethical approval for this study was waived by the medical research ethics committee of the University Medical Centre Utrecht.

### 2.2. Survey and Data Collection 

A questionnaire was developed to collect information on vaccination history, including adherence to the national immunization program and medically indicated vaccines. The patients were asked to complete it (Table S1). We also included the patient’s self-reported history of prior chickenpox and measles infection to assess whether there would have been an indication for additional serological examination. In addition to the patient-reported answers, information on vaccination history and prior history of chickenpox and measles were collected from the electronic patient file (EPF). Patient history of measles and chickenpox was assessed by serological tests or physician notes. Additionally, data on demographics, immunosuppressive condition and medication, and attending physician were collected from the EPF. 

### 2.3. Definitions 

According to the guidelines, the following vaccines were defined as medically indicated: annual vaccination against respiratory viruses (influenza and SARS-CoV-2) and pneumococcal, herpes zoster, and HPV vaccination [18,31,32,33,34,35,36,37,38]. In case of seronegativity for measles or varicella-zoster virus (VZV), measles-, mumps- and rubella (MMR) respectively VZV-vaccination were defined as medically indicated [18,31,32,33,34,35,36,37,38]. Vaccination coverage was defined as the number of patients vaccinated according to the recommendations in guidelines. If a national guideline was available for a specific patient category, vaccination coverage was scored according to the recommendations in the guideline. If no national guideline was available, European and American guidelines were used. Patients were considered as having had pneumococcal vaccination when one of the two vaccines (either polysaccharide or conjugate) was given. 

A patient was scored as ‘vaccinated according to the EPF’ when a prescription was present or information on vaccination was written down either in physicians’ notes or reports. 

Medical history of chickenpox or measles was scored as confirmed when documented in patients’ file or when serological screening was positive. In addition, a history of measles was scored as positive based on the patient’s age (born before 1965) or known measles vaccination history. In case of negative serology, a patient was considered as not having had chickenpox or measles. When there was no documentation or serological screening present, vaccination status was considered as unknown. 

### 2.4. Recommendations for Vaccination

Dutch, European (ESCMID), and American (ACIP) guidelines were consulted to assess guideline recommendations for medically indicated vaccines for ICPs [18,31,32,33,34,35,36,37,38]. According to the guidelines, pneumococcal vaccination was considered indicated for all ICPs regardless of age [33,34]. Because recommendations for vaccination with the inactivated herpes zoster vaccine (Shingrix) were included in guidelines at the start of this study, the indications for the vaccination adhered to were based in part on the previous recommendations for the live attenuated herpes zoster vaccine: indicated for patients with inflammatory auto-immune diseases and people living with HIV ≥ 50 years of age; and indicated regardless of age for all other ICPs [18,32,36]. HPV vaccination was considered indicated for all female ICPs ≤ 26 years of age according to the Dutch guidelines during the study period [18,35,37]. 

### 2.5. Data Synthesis and Statistical Analysis

Responses to the questionnaires and data collected from the EPF were processed and pseudonymously stored in Castor EDC (Electronic Data Capture) software, version 32.11. Statistical analyses were conducted using SPSS (version 29.0.2.0, IBM Corp., Armonk, NY, USA). Descriptive analyses summarized demographic data and clinical characteristics. Continuous variables are presented as the median with interquartile range, while categorical variables are presented as absolute numbers and frequencies (percentages). 

## 3. Results

During the study period in 2021–2022, in total, 116 ICPs admitted with COVID-19 were included in the study. One patient was excluded because of conflicting answers in the questionnaire, leaving 115 patients for analysis. The median age was 59 years (IQR 47, 66), and 61 patients (53%) were male (Table 1). The most frequent immunocompromising condition was solid organ transplantation (38%), followed by immune-mediated inflammatory diseases (IMIDs) (26%), and hematological malignancy (20%) (Table 1). Immunosuppressive medication was given for the following IMIDs: vasculitis (30%), rheumatoid arthritis (17%), systemic sclerosis (10%), systemic lupus erythematosus (7%), and others (37%), such as amyloidosis, IgG4 related disease, and IBD. No patients with HIV were included. 

### 3.1. Vaccination Coverage of Medically Indicated Vaccines

Patients’ self-reported vaccination coverage is as follows: 19% (22/115) for pneumococcal vaccination, 1% (1/106) for herpes zoster vaccination, and 40% (2/5) for HPV vaccination. Almost all patients (92%) were vaccinated for SARS-CoV-2, and 77% of the patients received the influenza vaccination annually. 

Because herpes zoster vaccination is reimbursed only for certain groups of ICPs in the Netherlands (patients with a hematopoietic stem cell transplantation, a solid organ transplantation, a malignancy, or with HIV), we investigated the effect of reimbursement on vaccination coverage. Therefore, we calculated the herpes zoster vaccination coverage for those who had an indication and were eligible for reimbursement (*n* = 80). Coverage was still low at 1% (1/80).

Cross-checking the EPF for vaccination rates showed that, in 21 patients (18%), a vaccination history was recorded. All of these patients received pneumococcal vaccination. No information was found in any of the eligible patients on herpes zoster or HPV vaccination. 

The low vaccination coverage limited the possibility of conducting additional analyses to examine the association between vaccination status and the underlying immunocompromising condition.

### 3.2. History of Varicella and Measles

Physicians were aware of patients’ varicella history in 70 (61%) patients. In 64 (91%) of these patients, history was known based on serological screening, and in 6 (9%) patients, it was known based on medical history. Of the 45 patients of whom physicians were not aware of patient varicella history, 27 (60%) patients could report a medical history of chickenpox. 

For only five (4%) patients, the physician had recorded measles infection status in the medical file; two patients were considered immune due to prior vaccination, and three patients were not protected based on negative serology. In the remaining patients (*n* = 110 (96%)), nothing was stated, but 58 (50%) can be considered immune due to natural infection based on year of birth. A total of 51 (44%) patients reported to have had measles. 

### 3.3. Relationship Between Vaccination Coverage of Different Vaccines 

Of the 27 patients who reported not being vaccinated for influenza, 19 (70%) had accepted COVID-19-vaccines. It follows that eight (30%) patients who did not receive the influenza vaccine were also not vaccinated against SARS-CoV-2, regardless of universal recommendations. Of these eight patients, one (13%) was reported to have received pneumococcal vaccination. Of the patients who had received pneumococcal vaccination (n = 22), 21 (96%) patients also had taken COVID-19 and influenza vaccinations.

## 4. Discussion 

This study shows that vaccination coverage for medically indicated vaccines in ICPs is low among our cohort of patients hospitalized with COVID-19 in the Netherlands. Respectively, 19%, 1%, and 40% of patients eligible for pneumococcal, herpes zoster, or HPV vaccination received the vaccine. On the contrary, vaccination coverage for COVID-19 and seasonal influenza was much higher at 92% and 77%, respectively. 

The vaccination rates we found are lower than those reported in previous studies in subgroups of ICPs. In two Canadian studies on patients with rheumatic conditions, pneumococcal vaccination rates for at least one polysaccharide or conjugate vaccine were 35.7% and 36.7%. Herpes zoster vaccination rates were 18.4% and 19% [24,39]. In IBD patients in Spain, pneumococcal vaccination coverage for receiving both polysaccharide and conjugate vaccines was 19% [23]. Although our cohort of patients should be considered more severely immunocompromised (98% of patients) and probably more vulnerable to infections, vaccination coverage was even lower. 

### 4.1. Determinants of Low Vaccination Coverage

Various factors may explain this low vaccination coverage. Vaccination recommendations are not always clear for healthcare providers, difficult to find, and scattered over many guidelines focusing on all specific groups of ICPs. More than 18 national and international guidelines are available on vaccination practices in ICPs [17,40,41,42]. Many subgroups of ICPs use similar immunosuppressive agents, but not all medical specialties align their recommendations to the same patient group from another medical specialty. Moreover, the strength of recommendations for vaccinations may be weak due to limited data on the effectiveness or immunogenicity of each subgroup of ICPs. Hence, these weak recommendations lead to provider hesitancy due to a perceived lack of benefit [25,43]. 

Until 2019, no Dutch national vaccination guideline was available for patients with chronic inflammatory auto-immune diseases that was endorsed by all relevant medical associations. Additionally, a national guideline for vaccinating patients with hematological diseases was only published in 2023. Clear guidelines may help reduce physicians’ knowledge gaps about vaccinations. Guideline-driven serologic screening and vaccination result in better insights into patients’ susceptibility and higher vaccination rates [44,45]. In addition, increased knowledge among physicians may improve patients’ willingness to get vaccinated as a physician’s recommendation is crucial for encouraging vaccine uptake [24,46]. 

Another barrier to vaccination is the reimbursement policy. An unclear reimbursement policy is seen as the biggest barrier for clinicians in recommending and administering vaccines [47,48]. In the Netherlands, varying reimbursement policies for different vaccines and groups of ICPs add complexity. Differences in vaccine administration between hospital and GP settings also create administrative barriers. These factors hinder the development of a uniform vaccination strategy for ICPs and negatively impact vaccination intentions and administration [49]. 

Finally, differences in vaccine administration may contribute to low vaccination coverage. In the Netherlands, vaccination care is complexly organized, which may contribute to low and slow uptake. Along with the various reimbursement schemes, the selection and administration processes differ for each vaccine. These differences in systems of vaccine administration also lead to unclear vaccination history, as there is no central registration system for tracking vaccine administration.

### 4.2. High Vaccination Uptake of COVID-19 and Influenza Vaccines

The determinants of vaccine uptake described above may explain a substantial part of why the uptake of COVID-19 and influenza vaccines is much higher than that of other vaccines. COVID-19 and influenza vaccines are, in the Netherlands, offered by national immunization programs and not by the attending physician. A prescription from the attending physician is thus not required for these vaccines, and vaccination coverage of these two vaccines is thereby less hampered by healthcare professional-related barriers or unclarity in the administration process. In addition, it is clear to both physicians and patients that these vaccines are reimbursed, thereby eliminating reimbursement-related concerns. 

Ultimately, it would have been valuable to further explore why patients reported not being vaccinated against pneumococci, herpes zoster, and HPV. However, such questions are difficult for patients to answer. If patients do not receive a vaccination invitation from their attending physician, they are unlikely to know the reason for not being vaccinated. Our review of electronic patient records indicates that vaccination is rarely discussed by physicians, which seems to be the primary reason for the low vaccination uptake.

### 4.3. Limitations and Strengths

One of the limitations of this study is that it is a single-center study with a relatively small sample size. Vaccination coverage, clinical practice, adherence to guidelines, and record keeping may be specific to this tertiary center. However, being a single-center study allows for a direct comparison of vaccination coverages. Additionally, as the results consistently show that most vaccines are not prescribed by physicians, we expect that expanding the sample size would not significantly affect the results or vaccination coverage.

Secondly, we calculated the HPV vaccination coverage in female ICPs as it was only recommended for female patients at the time of the study. However, since 2021, this vaccine has also been indicated for men, and these data are lacking in our study. 

In addition, our study only included ICPs who were hospitalized with COVID-19. The focus on COVID-19 may have reduced the perceived risk of other infections, and access to other preventive vaccines could have been limited during the pandemic. This may have contributed to lower vaccination coverage due to reduced access to vaccination services. 

On the contrary, the coverage for COVID-19 vaccination has given insight into the vaccination willingness of patients. In our cohort, coverage for COVID-19 vaccination was higher than the national average (92% versus 89.1%) [50].

The strength of this study lies in its focus on severely immunocompromised patients from various medical specialties. This approach provides insight into vaccination rates among those most in need and into vaccination practices across medical specialties.

## 5. Conclusions

In our cohort study, coverage of pneumococcal, herpes zoster, and HPV vaccination in moderate to severely immunocompromised patients was low. In contrast, vaccination rates with COVID-19 and influenza vaccines were high, showing that willingness for vaccination is present among patients. Identifying which factors contributed to high COVID-19 and influenza vaccines uptake can help to improve vaccination rates for the other recommended vaccines in ICPs. To enhance guideline implementation and increase vaccination rates, uniformity of vaccination recommendation, administration practices, and reimbursement policies are essential.

## Figures and Tables

**Table 1:** Patient characteristics.

Characteristics	*n* = 115
Age in years, median (IQR)	59 (47, 66)
Male, *n* (%)	61 (53%)
Duration of immunocompromised state	
<1 yr	17 (15%)
1–2 yrs	18 (16%)
2–5 yrs	26 (23%)
5–10 yrs	17 (15%)
>10 yrs	31 (27%)
Unknown	6 (5%)
Immunosuppressive conditions	
IMIDs for which immunosuppressive medication, *n* (%)	30 (26%)
Solid organ transplant, *n* (%)	44 (38%)
heart	4 (9%)
kidney	26 (59%)
lung	14 (32%)
Stem cell transplant, *n* (%)	11 (10%)
allogenic	8 (73%)
autologous	3 (27%)
Hematological malignancy, *n* (%)	23 (20%)
B-cell malignancy without chemo/immunotherapy	1 (4%)
B-cell malignancy with chemo/immunotherapy	17 (74%)
CAR T-cell therapy	5 (22%)
Solid malignancy, *n* (%)	1 (1%)
Primary immunodeficiency, *n* (%)	6 (5%)
CVID	4 (67%)
XLA	1 (17%)
CID	1 (17%)

Abbreviations: CAR: chimeric antigen receptor; CID: combined immunodeficiency; CVID: common variable immunodeficiency; IMIDs: immune-mediated inflammatory diseases; IQR: interquartile range; XLA: X-linked agammaglobulinemia.

## Data Availability

The datasets generated and/or analyzed during the current study are not publicly available due to privacy reasons but are available from the corresponding author upon reasonable request.

## Supplementary Materials

The following supporting information can be downloaded at: https://www.mdpi.com/article/10.3390/vaccines12121383/s1. Table S1: questionnaire.

## Abbreviations

ICPs: immunocompromised patients; HPV: human papillomavirus; RZV: recombinant zoster vaccine; IBD: inflammatory bowel disease; EPF: electronic patient file; VZV: varicella-zoster virus; IQR: interquartile range; IMID: immune-mediated inflammatory disease; CAR: chimeric antigen receptor; CID: combined immunodeficiency; CVID: common variable immunodeficiency; XLA: X-linked agammaglobulinemia.
