# Supplementary material for: Vaccination Coverage for Medically Indicated Vaccines in a Convenience Sample of Severely Immunocompromised Patients with COVID-19: An Observational Cohort Study
Source: Vaccines (Basel). 2024 Dec 9;12(12):1383. doi: 10.3390/vaccines12121383 (PMC11680350; doi:10.3390/vaccines12121383)
Supplement: Supplementary file 1 [file vaccines-12-01383-s001.zip › vaccines-3306213-supplementary.pdf]

Table S1: questionnaire

Vaccination history	Yes (%)	No (%)	Unknown (%)
• Did you receive pneumococcal vaccination?	19.1	67.0	13.9
• Did you receive herpes zoster vaccination?	.9	91.5	7.5
• Did you receive HPV vaccination?	40.0	60.0	-
• Did you receive COVID-19 vaccination?	92.2	7.8	-
• Do you receive seasonal influenza vaccination annually?	76.5	23.5	-
• Have you been vaccinated according to the national immunization program as a child?	87.0	8.7	4.3
**Previous infectious diseases**			
• Have you ever had chickenpox?	62.6	11.3	26.1
• Have you ever had measles?	44.3	28.7	27.0
